# Non-Coding RNA Sequencing of Equine Endometrium During Maternal Recognition of Pregnancy

**DOI:** 10.3390/genes10100821

**Published:** 2019-10-18

**Authors:** Kristin M. Klohonatz, Stephen J. Coleman, Ashley D. Cameron, Ann M. Hess, Kailee J. Reed, Angela Canovas, Juan F. Medrano, Alma D. Islas-Trejo, Ted Kalbfleisch, Gerrit J. Bouma, Jason E. Bruemmer

**Affiliations:** 1 Department of Animal Sciences, Colorado State University, Fort Collins, CO 80523, USA; kmk5057@gmail.com (K.M.K.); stephen.coleman@colostate.edu (S.J.C.); adcamero@gmail.com (A.D.C.); kailee.reed@colostate.edu (K.J.R.); 2 Department of Statistics and Bioinformatics, Colorado State University, Fort Collins, CO 80523, USA; ann.hess@colostate.edu; 3 Department of Animal Science, University of California Davis, Davis, CA 95616, USA; acanovas@uoguelph.ca (A.C.); jfmedrano@ucdavis.edu (J.F.M.); adislas@ucdavis.edu (A.D.I.-T.); 4 Department of Veterinary Science, Gluck Equine Research Center, University of Kentucky, Lexington, KY 40503, USA; ted.kalbfleisch@uky.edu; 5 Department of Biomedical Sciences, Animal Reproduction and Biotechnology Laboratory, Colorado State University, Fort Collins, CO 80523, USA; gerrit.bouma@colostate.edu; 6 Department of Animal Sciences, 259 Animal Sciences, 1171 Campus Delivery Colorado State University, Fort Collins, CO 80523, USA

**Keywords:** equine, pregnancy, maternal recognition of pregnancy, embryo, non-coding RNA, endometrium

## Abstract

Maternal recognition of pregnancy (MRP) in the mare is not well defined. In a non-pregnant mare, prostaglandin F_2α_ (PGF) is released on day 14 post-ovulation (PO) to cause luteal regression, resulting in loss of progesterone production. Equine MRP occurs prior to day 14 to halt PGF production. Studies have failed to identify a gene candidate for MRP, so attention has turned to small, non-coding RNAs. The objective of this study was to evaluate small RNA (<200 nucleotides) content in endometrium during MRP. Mares were used in a cross-over design with each having a pregnant and non-mated cycle. Each mare was randomly assigned to collection day 11 or 13 PO (*n* = 3/day) and endometrial biopsies were obtained. Total RNA was isolated and sequencing libraries were prepared using a small RNA library preparation kit and sequenced on a HiSeq 2000. EquCab3 was used as the reference genome and DESeq2 was used for statistical analysis. On day 11, 419 ncRNAs, representing miRNA, snRNA, snoRNA, scaRNA, and vaultRNA, were different between pregnancy statuses, but none on day 13. Equine endometrial ncRNAs with unknown structure and function were also identified. This study is the first to describe ncRNA transcriptome in equine endometrium. Identifying targets of these ncRNAs could lead to determining MRP.

## 1. Introduction

Early pregnancy in the mare is a process that is still not well defined. Specifically, the signal for maternal recognition of pregnancy (MRP) remains a mystery. The equine embryo does not attach to the endometrium until approximately day 35 post-ovulation (PO). Therefore, the communication between the embryo and the endometrium occurs without any attachment of the two. During the estrous cycle, if the endometrium fails to recognize a viable embryo, it produces prostaglandin F_2α_ (PGF) on day 14 PO to cause regression of the corpus luteum (CL) [1,2]. The corpus luteum is necessary during pregnancy to produce progesterone. There may be the presence of a conceptus, but this does not necessarily mean that the mare will be pregnant or undergo maternal recognition of pregnancy (such as with concomitant inflammation/aneuploidy) [3].

The hormonal profile of both pregnant and non-pregnant mares is the same until day 14 PO [2]. In the non-pregnant mare, when no embryo is recognized, oxytocin is produced by both the posterior pituitary gland and endometrium. Endometrial receptors bind the oxytocin, which ultimately causes the production and release of more oxytocin, resulting in the production of PGF [1].

In a pregnant mare, fertilization occurs in the oviduct and the conceptus enters the uterus on day 6 by releasing prostaglandin E_2_ (PGE) around day 4 [4]. When the embryo enters the uterus, it is surrounded by an acellular glycoprotein capsule, a feature that is unique to equine and rabbit embryos [5,6]. At this point, the embryo is mobilized throughout the entire uterus by uterine contractions [7]. This mobility is necessary in order to signal MRP [8,9]. Maximum mobility occurs between days 11–14, then ceases movement by day 16 when the embryo becomes fixed in a single location, but does not attach or invade [7]. Maternal recognition of pregnancy occurs during the time frame when the embryo is most mobile (days 11–14) [10]. It is thought that this embryo mobility impacts focal adhesion molecules on the endometrium, leading to MRP [11,12]. If a mare is pregnant, MRP occurs, and the endometrium will not produce or release PGF, therefore allowing the CL and its progesterone production to be sustained, indicating that MRP in the mare is antiluteolytic [7,10,13].

Previous experiments have tested the signals for MRP in other species, such as estradiol (pigs) and Interferon tau (cows), on the mare, but these result in no changes in equine luteal function [14,15]. Studies have progressed to evaluating gene expression within the endometrium, but no obvious gene candidates have been uncovered [11,16,17,18]. Recent studies in our laboratory have identified that the content, specifically miRNAs, of serum exosomes differ between pregnant and non-pregnant mares during MRP [19]. The source and function of these miRNAs are unknown, but due to their difference based on pregnancy status it could be hypothesized that they are of endometrial origin. A more recent study in our lab utilized RNA sequencing to evaluate coding RNA in equine endometrium during maternal recognition of pregnancy based upon pregnancy status [12]. An interesting result was that what appeared to be a large number of transcripts underwent alternative splicing during this time frame. These studies raised interest in evaluating the small non-coding RNA content in endometrium during MRP.

Small non-coding RNAs (ncRNAs), which are less than 200 nucleotides long, do not code for protein and are typically involved in the regulation of RNA processing and function [20]. We hypothesized that endometrial ncRNA content would be altered in relation to pregnancy status. The objective of this study was to identify small ncRNAs, focusing mainly on miRNAs, and their temporal expression changes in equine endometrium during maternal recognition of pregnancy via RNA sequencing.

## 2. Materials and Methods 

### 2.1. Care and Management of Mares

Horse use was approved by the Colorado State University Institutional Animal Care and Use Committee. Mares (*n* = 6 total) were housed in group pens at the Colorado State University Bud and Jo Adams Equine Reproduction Laboratory (Fort Collins, CO, USA). Horses were maintained on a dry lot and fed a grass–alfalfa hay mix with free choice mineral and salt supplement. Mares were used in a paired, cross-over design where each mare had a pregnant and non-pregnant (non-mated) cycle for their specified collection day. Mares were randomly assigned to a collection day (day 11 or 13 post-ovulation, *n* = 3 per day). Mares were monitored via transrectal palpation and ultrasonography to track their follicular development every other day. To obtain samples from a pregnant mare, once a follicle reached 35 mm in diameter, or larger, the mare was inseminated with at least 500 × 10^6^ progressively motile sperm from a stallion. Different stallions were utilized, but all with proven fertility. Mares were then monitored via transrectal ultrasonography every day and inseminated every other day until ovulation (day 0).

On the mare’s assigned day, she was evaluated via transrectal ultrasonography to confirm pregnancy status by visualization of an embryonic vesicle. Endometrial samples were obtained via a trans-cervical biopsy punch, followed by a terminal uterine lavage to again confirm pregnancy status [21]. After endometrial sample collection, the mare received a luteolytic dose of PGF (Estrumate, Merck Animal Health, 250 mcg per dose) and the subsequent estrous cycle was used for the non-pregnant (non-mated) cycle. After endometrial sample collection, the sample was rinsed in DPBS/Modified 1X (Hyclone Laboratories, Logan, UT) and stored at −80 °C until further analysis.

### 2.2. RNA Isolation and Quantification

Total RNA from the endometrium was extracted using the mirVana miRNA Isolation kit (Invitrogen, Carlsbad, CA, USA) at the University of California-Davis. Briefly, samples were homogenized in lysis/binding buffer. MiRNA homogenate additive was added to the lysate and incubated for 10 min on ice. Acid-Phenol:Chloroform was added to the lysate, vortexed, and centrifuged at 10,000 × *g* for 5 min. The sample was then separated into three distinct phases (RNA, DNA, and protein). The top, aqueous RNA phase was transferred to a new 1.7 mL tube for RNA purification. Ethanol was added to the RNA phase solution and then passed through a filter provided by Invitrogen. The filters were then washed with miRNA wash solution and eluted in the Elution Solution. RNA purity and quantification were assessed using the NanoDrop Spectrophotometer ND-1000 (Thermo Scientific, Wilmington, DE). Samples were used for PCR analysis if they had 260/280 and 260/230 values above 1.7.

### 2.3. RNA Sequencing

Libraries were prepared at the University of California-Davis using the Illumina TruSeq Small RNA Library Preparation kit (Illumina, San Diego, CA, USA) and 1 μg of Total RNA from each sample, following the manufacturer’s protocol. Briefly, adapters were ligated, and samples were reverse transcribed to cDNA. Each sample was amplified with a specified barcoded PCR primer for identification purposes. Samples were pooled and run out on a DNA gel, and the region of 147–157 nucleotides was excised and isolated from the gel. This represented the small RNA that was to be sequenced. After gel extraction, the samples were suspended in 10 mM Tris-HCl, pH 8.5. Libraries were evaluated for quality with a Bioanalyzer (Agilent, Santa Clara, CA) and sent to the University of California-Berkeley for sequencing using a HiSeq 2000 sequence analyzer (Illumina, San Diego, CA, USA). Sequences are available in the NCBI sequence read archive under BioProject PRJNA545717.

### 2.4. Bioinformatic Analysis

Bioinformatic analysis was performed on the Galaxy web platform [22] and used the public server at usegalaxy.org. Sequence quality was assessed by FastQC, and results were aggregated and evaluated using MultiQC [23,24]. Trimmomatic was utilized to remove the adapter sequence and low-quality sequence [25]. Reads were aligned to EquCab3.0 ncRNA FASTA from Ensembl (version 96) and transcripts quantified using Salmon [26,27,28]. Count data (number of reads from Salmon) were analyzed within each day, comparing samples from pregnant mares to non-pregnant mares utilizing DESeq2 within R [29]. To be considered for analysis, reads were present in at least two out of the three replicates in at least one of the two groups (P + or NP). Data were normalized internally using DESeq2’s median of ratios method. Benjamini–Hochberg false discovery rate adjustment was used. Significance was assessed at *p* ≤ 0.05.

## 3. Results

### 3.1. Sequencing Results

Quality control assessment indicated that reads had a high adapter content. Based upon these results, the 50 base pair reads were trimmed to 23 base pairs for all samples. Samples generated, on average, 10,288,100 reads (ranging from 2,713,658 to 17,261,023). 

### 3.2. Small RNA Transcript Analysis

**Figure 1:**
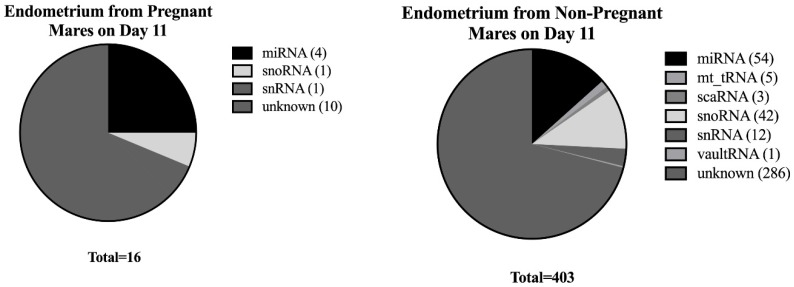
Distribution of differentially expressed families of ncRNA in endometrial samples on day 11 from both pregnant and non-pregnant mares. This figure represents the families of ncRNA differentially expressed (*p* ≤ 0.05) in endometrial samples on day 11.

Overall, there were 8370 non-coding RNAs identified in the equine endometrium on day 11 and 3984 on day 13. Of the ncRNA identified in the endometrium, 3653 were present on both days 11 and 13. On day 11, there were 419 differentially expressed small non-coding RNAs (ncRNAs; *p* ≤ 0.05) between samples from pregnant and non-pregnant mares. Only 16 of these ncRNAs were more abundant in samples from pregnant mares (3.8%), and 403 were more abundant in samples from non-pregnant mares (96.2%). These 419 ncRNAs represented multiple families of ncRNA, including miRNA (microRNA), snRNA (small nuclear RNA), snoRNA (small nucleolar RNA), and scaRNA (small Cajal body-specific RNA; Figure 1). Only 123 transcripts were identified as specific ncRNAs; the remaining 296 were identified as ncRNAs in the horse, but with unknown structure or function by Ensembl (version 96). The largest family of known ncRNA represented in this study was our focus, miRNA (58 out of 419 differentially expressed ncRNA). Table 1 contains the top 20 most significant known ncRNA in our dataset. Supplemental Table S1 contains the complete list of differentially expressed ncRNAs on day 11.

Of the 419 significant differentially expressed ncRNAs on day 11, 7 were present only in samples from pregnant mares (2 miRNAs and 5 unknown ncRNAs) and 175 were present only in samples from non-pregnant mares (2 miRNAs, 2 snRNAs, 1 snoRNA, and 170 unknown ncRNAs). On day 13, there were no differences in ncRNA expression between the two sample groups. 

This table breaks down the significant (*p* ≤ 0.05) ncRNAs with known structure and function in the endometrium on day 11 ranked based upon significance. The average represents the average number of reads determined during Salmon analysis. Non-coding RNA names bolded indicate they were more highly expressed in samples from pregnant mares.

## 4. Discussion

These are the first sequence data to investigate small ncRNA in equine endometrium during maternal recognition of pregnancy. Non-coding RNA transcripts, like miRNA, were once considered part of the genome’s “junk” DNA, which was believed to be non-functional in terms of transcription. Even though they do not code for proteins, miRNAs have critical roles as post-transcriptional regulators of protein-coding gene expression impacting the mRNA abundance of up to 60% of protein-coding genes in mammals [30]. This study identified many different families of ncRNA, including miRNA, snRNA, and snoRNA. Each of these families plays an essential role in the post-transcriptional regulation of gene expression. 

MicroRNAs are short single-stranded RNAs, approximately 22 nucleotides in length [31], that represent a highly conserved subset of ncRNA across species. The majority of miRNAs are transcribed by RNA polymerase II as primary-miRNA (pri-miRNA) transcripts [32]. Drosha and DGCR8 then cleave the pri-miRNA into 70 nucleotide stem–loop precursor miRNAs (pre-miRNA) while the RNA is still in the nucleus [33]. The pre-miRNA is then exported out of the nucleus into the cytoplasm, where it is cleaved by the Dicer complex [33]. The Dicer complex cleaves the stem–loop into double-stranded miRNA, which is then loaded onto an AGO protein [34]. The AGO protein unwinds the duplex and loads the guide strand of the mature miRNA onto the RNA-induced silencing complex (RISC) [34]. This guide miRNA then guides RISC to the target mRNA [34]. In many instances, one miRNA binds multiple different mRNAs due to imperfect complementary base pairing to the target sites in the 3’ untranslated regions [35]. This binding destabilizes the mRNAs and usually results in the degradation of the mRNAs. 

In this study, miRNAs were one of the top families of ncRNA identified in the endometrium. Endometrium on day 11 contained 58 significantly differentially expressed miRNAs. Interestingly, the majority (54 out of 58) of miRNAs were more highly abundant in samples from non-pregnant mares. Of the four miRNAs that were more abundant in samples from pregnant mares, all have been previously described. Eca-mir-8986b has not been identified in equine endometrium, but it is present in microvesicles secreted from amniotic mesenchymal cells [36]. Microvesicles act as a vehicle for transport of nucleic acids, lipids, and proteins from cell to cell in a form of communication [37,38]. Eca-mir-1291a has previously been described in the equine epididymis [39]. Beyond this identification, eca-mir-1291a has not been identified anywhere else in the body in both horse and other species. Eca-mir-19a has previously been identified in extracellular vesicles in bovine antral follicles [40]. Mir-19a is also an inhibitor of suppressors of cytokine signaling (SOCS), which inhibit the Janus kinase–signal transducer and activator of transcription (JAK–STAT) pathway [41,42,43]. JAK–STAT signaling mediates cell proliferation and inflammation [44]. While it has not been identified in equine endometrium, eca-mir-539 is enriched in microvesicles in equine follicular fluid [45]. It is also present in the equine epididymis [39].

There were 54 miRNAs that were more abundant in endometrial samples from non-pregnant mares. Out of the 54, 3 had an unknown function and 31 were equivalent to human miRNAs. Those 31 miRNAs were evaluated with DIANAmiR Path to determine KEGG pathways they were targeting [46]. The top pathways protein processing in endoplasmic reticulum, adherens junctions, hippo signaling pathway, and the cell cycle. Table 2 contains the top 20 processes and their *p*-values. Supplemental Table S2 contains all of gene ontology categories targeted.

In a previous study, we evaluated serum exosome miRNA content in relation to pregnancy status and identified differentially expressed miRNAs between samples from pregnant and non-pregnant mares [19]. We compared those miRNAs to the miRNAs identified in the present study. While the serum exosome miRNAs are present in the endometrium on either both days (11 and 13) or just day 11 or day 13, none were significantly more expressed in samples from either pregnant or non-pregnant mares. This leads to the question of the source of the serum miRNA, and it is undetermined whether the endometrial miRNAs are from the serum or are the serum exosomal miRNAs from the endometrium. 

Another family of ncRNA that was represented in this study was small nuclear RNAs or snRNAs. They have well-defined functions in mRNA splicing and 3′-end formation [47]. Small nuclear RNAs are approximately 150 nucleotides in length and are transcribed by RNA polymerase II or III [47]. They are also components of the spliceosome to participate in pre-mRNA processing [48]. In this study, snRNAs represented 13 of the significant ncRNA in the endometrium on day 11. Only one of these ncRNAs were more abundant in samples from pregnant mares, and the remaining 11 were more abundant in samples from non-pregnant mares. The snRNA that is more abundant in samples from pregnant mares is in the family of U5 snRNA [49]. U5 snRNAs are involved in both the first and second catalytic steps in pre-mRNA processing [50]. Of the 12 snRNAs that are more abundant in samples from non-pregnant mares, 7 are in the U6 family, 3 are in the U1 family, and 2 are in the U2 family. U6 snRNAs are involved in the 5’ splice site choice [51]. U1 snRNAs function to protect pre-mRNAs from premature cleavage [52]. These snRNAs may be responsible for the alternative splicing that was identified in our previous study [12].

The next largest family of ncRNA identified in this study was small nucleolar RNAs, or snoRNAs. Small nucleolar RNAs are mainly involved in controlling ribosome biogenesis by guiding the modification or processing of pre-rRNA [53]. There are two main classes of snoRNA, C/D box and H/ACA box. C/D box snoRNAs are primarily associated with methylation modifications, and H/ACA box snoRNAs are primarily associated with pseudouridylation [53]. All small nucleolar RNAs can also be processed into smaller fragments that participate in RNAi [54]. All snoRNAs identified in this dataset were more abundant in samples from non-pregnant mares. Also identified in this dataset were scaRNAs (small Cajal body-specific RNA), which are a class of snoRNA. Small Cajal body-specific RNAs are the modification guide RNAs for spliceosomal snRNAs [55].

In conclusion, this project was the first study to describe the small ncRNA transcriptome in equine endometrium. There were many novel ncRNA transcripts identified that need to be evaluated further. While differential expression of ncRNA was identified in samples from day 11, by day 13, there no transcripts with differential expression between samples from pregnant and non-pregnant mares. This suggests that by day 13, most mRNA control from ncRNAs associated with MRP may be concluded. Small RNAs have a robust role in modifying transcription and understanding these ncRNAs and their targets further could lead to identifying the mechanism for maternal recognition of pregnancy in the horse.

## Figures and Tables

**Table 1:** Top 20 differentially expressed non-coding RNA in the endometrium on day 11 with known structure.

Feature ID	ncRNA Name	ncRNA Description	Day 11P+ Average	Day 11NP Average	Day 11 *p*-Value
**ENSECAT00000029757**	U109	snoRNA	1	71,765	2.1E-07
**ENSECAT00000028132**	U1	snRNA	0	21,351	5.4E-07
**ENSECAT00000032319**	eca-mir-9109	miRNA	9	15,762	6.3E-07
ENSECAT00000055936	eca-mir-8986b	miRNA	448	0	1.1E-06
**ENSECAT00000027602**	eca-mir-323	miRNA	11	39,904	1.2E-06
**ENSECAT00000027815**	SNORA69	snoRNA	49	36,277	6.7E-06
**ENSECAT00000028354**	MIR15B	miRNA	81	39,222	9.2E-06
**ENSECAT00000032694**	U6	snRNA	0	4866	1.5E-05
**ENSECAT00000027790**	MIR137	miRNA	2	2916	1.6E-05
**ENSECAT00000027318**	SNORA2B	snoRNA	4	5177	1.7E-05
**ENSECAT00000028446**	SNORD65	snoRNA	20	9278	1.7E-05
**ENSECAT00000029752**	SCARNA17	scaRNA	16	5915	2.3E-05
**ENSECAT00000029923**	eca-mir-9080	miRNA	13	5718	4.4E-05
**ENSECAT00000028257**	SNORA71	snoRNA	22	8449	4.4E-05
**ENSECAT00000027964**	eca-mir-652	miRNA	24	9678	4.4E-05
**ENSECAT00000028540**	SNORA12	snoRNA	2	1924	4.6E-05
**ENSECAT00000028054**	MIR1306	miRNA	15	4741	5.1E-05
**ENSECAT00000027873**	eca-mir-582	miRNA	3	2342	1.0E-04
**ENSECAT00000028318**	SNORA36A	snoRNA	48	12,262	1.3E-04
**ENSECAT00000027279**	SNORD59A	snoRNA	246	38,612	1.8E-04

This table breaks down the significant (*p* ≤ 0.05) ncRNA with known structure and function in the endometrium on day 11 ranked based upon significance. The average represents the average number of reads determined during Salmon analysis. Non-coding RNA names bolded indicate they were more highly expressed in samples from pregnant mares.

**Table 2:** Twenty most significant KEGG Pathways.

KEGG Pathway	*p*-Value
Protein processing in endoplasmic reticulum	1.54E-12
Proteoglycans in cancer	3.66E-12
Adherens junction	1.97E-11
Viral carcinogenesis	1.97E-11
Hippo signaling pathway	2.06E-08
p53 signaling pathway	3.44E-07
Cell cycle	3.74E-07
Renal cell carcinoma	4.62E-07
Pancreatic cancer	1.06E-06
Ubiquitin mediated proteolysis	1.54E-06
Neurotrophin signaling pathway	1.54E-06
TGF-beta signaling pathway	2.25E-06
Prostate cancer	2.25E-06
Epstein–Barr virus infection	2.25E-06
Pathways in cancer	2.25E-06
Glioma	2.77E-06
mRNA surveillance pathway	4.95E-06
Chronic myeloid leukemia	5.02E-06
Hepatitis B	5.02E-06
Endocytosis	6.27E-06

## Supplementary Materials

The following are available online at https://www.mdpi.com/2073-4425/10/10/821/s1: Table S1: All significant ncRNAs (*p* ≤ 0.05; known and unknown) in equine endometrium from pregnant and non-pregnant mares on day 11 post-ovulation; Table S2: All Kegg pathways associated with the differentially expressed miRNA on day 11 that were more abundant in samples from non-pregnant mares.
